# Transcription Factor TaWRKY51 Is a Positive Regulator in Root Architecture and Grain Yield Contributing Traits

**DOI:** 10.3389/fpls.2021.734614

**Published:** 2021-10-21

**Authors:** Yuying Li, Yanfei Zhang, Chaonan Li, Xin Chen, Lili Yang, Jie Zhang, Jingyi Wang, Long Li, Matthew P. Reynolds, Ruilian Jing, Xinguo Mao, Chenyang Wang

**Affiliations:** ^1^College of Agronomy, Henan Agricultural University, Zhengzhou, China; ^2^Institute of Crop Sciences, Chinese Academy of Agricultural Sciences, Beijing, China; ^3^International Maize and Wheat Improvement Center, Texcoco, Mexico

**Keywords:** WRKY transcription factor, agronomic trait, association analysis, haplotype, functional marker

## Abstract

Wheat is one of the staple food crops. The utilization of elite genetic resources to develop resource-efficient wheat varieties is an effective approach to deal with the challenges of climate change and population growth. WRKY transcription factors (TFs) are multifaceted regulators of plant growth and development and response to environmental stress. The previous studies have shown that TaWRKY51 positively regulates the development of lateral roots, while its roles in agronomic trait development are not clear, and there is no functional marker for molecular breeding. To bridge the gap, we cloned the three members of *TaWRKY51* and found they were highly expressed in the roots and flag leaves at the flowering stage and were induced by the multiple abiotic stresses and phytohormones. The highest expression level was observed in *TaWRKY51-2D*, followed by *TaWRKY51-2A* and *-2B*. The two haplotypes/alleles for each member were identified in the natural populations, and functional markers were developed accordingly. The association assays revealed that *Hap*-2A-I was an elite haplotype for the large spike, *Hap*-2B-II and allele-G were favorable haplotypes/alleles for long root. However, only *Hap*-2A-I was selected for wheat breeding in China. The results of transgenic experiments showed that the rice lines overexpressing *TaWRKY51* had large panicle, high thousand-grain-weight, and more crown and lateral roots, which further confirmed the results of association analysis. In short, *TaWRKY51* is a positive regulator of the root architecture and grain yield (GY) contributing traits. The elite gene resources and functional markers may be utilized in the marker-assisted selection for high-yield breeding in wheat.

## Introduction

Common wheat (*Triticum aestivum* L.) as one of the staple food crops, plays important role in ensuring food security globally, providing about 20% of daily proteins and calories (Shiferaw et al., 2013). Great challenges are being imposed on wheat production in the scenario of climate change and a fast-growing population. The exploration and utilization of resource-efficient genes in wheat breeding is an economical and effective approach to resolve the problem. Roots play a key role in foraging for water and nutrients in the soil and perceiving environmental stimuli. The wheat root system is primarily composed of coarse and fine roots. The fine roots, including lateral root and root hairs, are more effective in extracting water and nutrients from the soil due to a larger surface area (Ahmed et al., 2016). However, very few genes or allelic variants have been identified for lateral root development in cereals (Yu et al., 2016).

The WRKY transcription factor (TF) is a large family of plant-specific regulatory proteins (Guo et al., 2012). The WRKY TFs are named by the presence of one or two highly conserved WRKY domains (WDs), including a conserved WRKYGQK motif at the N-terminus and a C_2_H_2_ zinc-finger structure at the C-terminus (Rushton et al., 2010). The WRKY family members are divided into three groups (I, II, and III) based on the number of WD and the characteristics of zinc-finger motifs (Eulgem et al., 2000). Group I typically has two WDs, whereas Group II and III are featured by a single WD. WRKY TFs regulate gene expression by exclusively binding to the W-box (TTGACC/T) present in the promoter region of downstream genes (Bakshi and Oelmuller, 2014). The WRKY TF was first identified in sweet potatoes (Ishiguro and Nakamura, 1994), and numerous WRKY members have been characterized in various plant species (Sun et al., 2003).

Accumulated data have documented that WRKY TFs are multifunctional regulators in plant growth and development, including seed development and germination (Raineri et al., 2016), stem elongation (Zhang et al., 2011; Yu et al., 2012), embryogenesis (Lagace and Matton, 2004; Jimmy and Babu, 2015), trichome development (Johnson et al., 2002), and senescence (Ricachenevsky et al., 2010; Sakuraba et al., 2016). Several WRKY TFs are involved in root development. For instance, AtWRKY46 modulates the development of lateral roots in Arabidopsis under osmotic/salt stress conditions (Ding et al., 2015). AtWRKY75 is implicated in the response to nutrient starvation and regulating the root architecture development (Devaiah et al., 2007). CbNN1 positively regulates the initiation of adventitious root in *Catalpa Scop* (Wang et al., 2019). In wheat, a few WRKY TFs are identified as positive regulators for root development. The overexpression of *TaWRKY1, TaWRKY10, TaWRKY33, TaWRKY46*, and *TaWRKY93* leads to the long roots and enhanced tolerance to abiotic stress (Wang et al., 2013; Ding et al., 2015; Qin et al., 2015; He et al., 2016). TaWRKY51 promotes lateral root formation by negatively regulating the ethylene biosynthesis in wheat (Hu et al., 2018).

A marker-assisted selection (MAS) is a key technology in precision crop breeding (Bertrand et al., 2008), which is not directly affected by the environment and can accurately select multiple target genes simultaneously in early generations (assuming close linkage to operative alleles), thus, potentially reducing the need for field phenotyping and breeding cycle length. At the same time, MAS can overcome the difficulties in the identification and application of recessive genes, therefore greatly improving the accuracy and efficiency of crop breeding (Sharma and Punya, 2020). For those reasons, MAS was fully expected to accelerate the breeding gains. However, MAS has not played such expected roles in wheat breeding especially for the complex traits because of the availability of functional markers.

The role of *TaWRKY51* in lateral root development has been functionally characterized, but the functions in developing agronomic traits remain unclear. To facilitate its application in crop breeding, its roles in agronomic trait development and nucleotide sequence polymorphisms were characterized. Moreover, favorable haplotypes were identified, and their geographic distributions were investigated in both the landraces and modern varieties in China. And the results showed that *TaWRKY51* is a positive regulator in the development of root architecture and grain yield contributing traits. This study provides valuable gene resources and functional markers for wheat molecular breeding.

## Materials and Methods

### Plant Materials and Growing Environments

In this study, 36 wheat accessions, selected from 262 accessions based on the DNA polymorphisms and population structures evaluated with 209 SSR markers, were utilized to analyze the nucleotide sequence polymorphisms in the target genes. Three wheat natural populations (NPs) were used for various purposes in the current research. The NP1 that consisted of 323 accessions was employed for the association analysis. The accessions were mainly released in the Northern winter wheat and Yellow and Huai River valley facultative wheat zones (Miao et al., 2017). The NP2 primarily consisted of landraces from the Chinese wheat mini-core collection representing more than 70% of the genetic diversity of the total Chinese germplasm collection. The NP3 was originated from the Chinese wheat core collection (Hao et al., 2011) and was used to identify the haplotypes and their geographic distributions of *TaWRKY51s*.

The NP1 was grown in the 16 environments (year × site × stress combinations) at the experimental station Champing (116°13′E, 40°13′N), Beijing in 2016 and 2017, and Shunyi (116°56′E, 40'23′N), Beijing during 2015–2017 cropping seasons. The field experiments were conducted under four regimes, such as non-stressed (NS), drought-stressed (DS), heat-stressed (HS), and combined drought and heat stressed (DHS) conditions. Each experimental plot consisted of four rows of 2-m long and 30 cm apart. Forty seeds were sown in each row at the end of September and harvested in late June of next year. The NS plots were irrigated with 750 m^3^ ha^−1^ (75 mm) water at each of the pre-overwintering, flowering, and grain filling stages, while the DS and DHS plots were rain-fed. The total precipitation during the growing seasons was 161, 173, and 127 mm in the years 2015, 2016, and 2017, respectively. The agronomic traits, such as plant height, peduncle length, spike per plant, spike length, spikelet number per spike, grain number per spike, and thousand-grain weight (TGW), were measured.

To probe whether the target gene is involved in the development of the root system in wheat. The root traits of NP1 were measured at different developmental stages. At the seedling stage, the plants were grown in the gel chambers, and the root traits including root number (RN), root length, and root angle were measured as described (Liu et al., 2013). To identify the root traits at the tillering, booting, and mid-grain-filling stages, the wheat seeds were sown in condensed soil columns coated with polyethylene tubes in mid-October, which were placed in the polyvinyl chloride (PVC) pipes before sowing. The data of root depth and root dry weight were collected at dedicated developmental stages as previously described (Li et al., 2019).

A wheat cultivar Hanxuan 10 with prominent drought tolerance was used for the gene cloning, genomic sequence isolation, and gene expression pattern assays. The rice variety Kitaake was used for the transgenic experiments. Three T3 homozygous transgenic rice lines with various expression levels of *TaWRKY51* were selected for phenotyping.

### Subcellular Localization of TaWRKY51

The open reading frame (ORF) of *TaWRKY51* was fused upstream of green fluorescent protein (GFP) in pJIT163-GFP expression vector driven by the cauliflower mosaic virus 35S promoter to construct 35S::TaWRKY51-GFP fusion protein. The restriction sites were added to the 5′ and 3′ ends of the coding region by PCR. The oligonucleotides for fusion GFP subcloning are listed in Table 1. A PCR product was digested with the relevant restriction endonucleases and then ligated with pJIT163-GFP plasmid cutting with proper enzymes to create a recombinant construct expressing TaWRKY51-GFP protein. The positive plasmids were confirmed by sequencing. The constructs were transformed into wheat protoplast cells by polyethylene glycol (PEG)-mediated method. After incubation at 25°C in dark for 36–48 h, fluorescence was observed using a laser scanning confocal microscope (Leica TCSNT, Germany). The GFP auto-fluorescence was collected in the range of 500–570 nm wavelength.

**Table 1:** The primers used in the current study for various purposes.

**Primer**	**Primer sequence (5^**′**^-3^**′**^)**	**Purpose**
GAsF	GAGCACCTATCTTATCTTGGGAG	Isolating the genomic fragment of *TaWRKY51-2A*
GAsR	GTCACGAAGGGAACATGGTC	
GBsF	CGGTAAGCCCGTGTCTCTG	Isolating the genomic fragment of *TaWRKY51-2B*
GBsR	TAAACTGATGCGGCCAAAAG	
GDsF	AGCACCTATCTTATCTTGGGAG	Isolating the genomic fragment of *TaWRKY51-2D*
GDsR	CTAAGTTGAATTAACATGGCGA	
PAsF	TCTGTGTTAGAGTCTCCGAGCGT	Amplifying the promoter region of *TaWRKY51-2A*
PBsF	GCAACAGGCAATTAACGTAGTGAC	Amplifying the promoter region of *TaWRKY51-2B*
PDsF	CTGTGTTAGTGTCTTCGGGCG	Amplifying the promoter region of *TaWRKY51-2D*
PsR1	TCCTCCAATCAGATCCATGGTC	Amplifying the promoter region of *TaWRKY51-2B*
PsR2	CCCTCCCGTATCCTCCAATC	Amplifying the promoter regions of *TaWRKY51-2A* and *-2D*
Sub-F	CT*ggtacc*ATGGAGCAGAAACTCATCTCTGAAGAGGATATGATGACCATGGATCT (*Kpn* I)	Subcloning *TaWRKY51* into binary vector pCAMBIA1391
Sub-R	CT*actagt* TCAGAGCTCCGGGAGCGGGGCGAG (*Spe* I)	
J163-F	AG*gagctc*ATGATGACCATGGATCT (*Sac* I)	Constructing subcellular localization vector
J163-R	AG*agatct*GAGCTCCGGGAGCGGGGCGAG (*Bgl*II)	
M13F	TGTAAAACGACGGCCAGT	Sequencing primers for *pEASY*-Blunt vectr
M13R	CAGGAAACAGCTATGACC	
APseqR1	GTGACCCACCTGTCAGAGACT	Sequencing the promoter of *TaWRKY51-2A*
BSeq F1	AAGGTGTACCAATTCCATCAT	Sequencing the promoter of *TaWRKY51-2B*
BSeq F2	TAGTTAGGAGTAGAGTTTGTCAT	
DSeq F1	CTTTGCTTTGTTGTTTCTTCATTC	Sequencing the promoter of *TaWRKY51-2D*
DSeq F2	CGGCAGAACTGGAGGAGGTGTATC	
DSeq R1	GGTGCTGTGCTGGTCTCG	
qRT-AF	TTAATTCAACTTAGCTTATG	Determining the expression of *TaWRKY51-2A*
qRT-AR	TCAAAGCTTCCAACTACACA	
qRT-BF	TGTTAATTCAACTTAGCTTG	Determining the expression of *TaWRKY51-2B*
qRT-BR	CATATTTTTCTTTCTCCTTC	
qRT-DF	GCCATTACCGTCGTCCTCGC	Determining the expression of *TaWRKY51-2D*
qRT-DR	AAGCTTCCAACTACACTTGC	
TaActin-qF	CTCCCTCACAACAACAACCGC	Quantifying gene expression in wheat
TaActin-qR	TACCAGGAACTTCCATACCAAC	
OsTublin-qF	TGAGGACTGGTGCTTACCGC	Quantifying gene expression in rice
OsTublin-qR	GCACCATCAAACCTCAGGGA	
AS-F	CTCCCTTCTTGGTTGGTGGG	Marker development for *TaWRKY51-2A*
AS-R	GGAGAAGAAACAATTCAGGCG	
B-Hpa11F	TGGCCGCTGCCACTGCTC	CAPS marker development for *TaWRKY51-2B*
B-Hpa11R	CGGTCCCATTCATTCATTCTTT	
DGMF	AATAAATTTGTGACTTTCGCAGAAG	CAPS marker development for *TaWRKY51-2D*
DGMR	TTTTTTAACCTCGTCCTGGCA	

### Quantitative Real-Time PCR (qRT-PCR) of *TaWRKY51*

For tissue-specific gene expression assay, the wheat tissues were harvested at the booting and flowering stage and stored in −80°C freezer for RNA extraction after freezing in liquid nitrogen. To analyze the expression of the target gene upon environmental stimuli and phytohormones, the uniform wheat seeds were germinated in ddH_2_O for 2 days; then, the seeds with buds of the same size were transferred to the plastic baskets, which were placed in shallow trays filled with water and cultured in a growth chamber (20 ± 1°C, 16 h light/8 h dark cycle). The two-leaf seedlings were exposed to PEG-6000 (−0.5 MPa), 250 mM NaCl, low temperature (4°C), heat (34°C), 50 μM methyl jasmonate (MeJA), 50 μM indole-3-acetic acid (IAA), and 50 μM abscisic acid (ABA) as described (Mao et al., 2010). The seedling leaves and roots were collected separately at 0, 0.5, 1, 2, 3, 6, 12, 24, 48, and 72 h following the treatments, immediately frozen in liquid nitrogen and stored at −80°C for RNA extraction. Five plants were harvested for each replicate, and the three replicates were set for each treatment. The samples harvested at 0 h were used as the control for the corresponding treatments.

Total RNA was extracted from the wheat tissues using a TRIZOL reagent (ThermoFisher Scientific, Hong Kong). First-strand cDNA was synthesized with a TIANScript cDNA First-Strand Synthesis Kit (TIANGEN, Beijing, China) following the instructions of the manufacturer. Then, the quantitative real-time PCR **(**qRT-PCR) was performed three times with an ABI PRISM® 7900 system using the SYBR Green PCR Master Mix kit (TaKaRa Biotechnology, Japan) according to the instructions of the manufacturer. The transcript of the *TaActin* gene was used to quantify the relative transcription levels in wheat (Table 1). The relative gene expression levels were estimated by the 2^−Δ*CT*^ method (Livak and Schmittgen, 2001). For transgenic rice, the transcript of *OsTublin* was used to quantify the expression levels of *TaWRKY51* in transgenic rice plants (Table 1).

### Generation and Phenotyping of Transgenic Rice

The cDNA sequence of *TaWRKY51* was obtained by sequencing a wheat full-length cDNA library. The ORF of *TaWRKY51* was amplified using *pfu* DNA polymerase with Hanxuan 10 cDNA, two endonuclease enzyme cutting sites of *Kpn* I and *Spe* I were added to the up- and down-stream of the ORF, respectively (Table 1), and a 30-bp MYC-tag sequence was added between *Kpn* I site and the first ATG of *TaWRKY51* and then, sub-cloned into binary vector pCAMBIA1391 cut with proper enzymes. After sequencing, the positive construct was transformed into agrobacterium strain EHA105 and transferred into wild-type (WT) rice. The positive transgenic plants were initially identified by PCR and then followed by sequencing.

Only T2 transgenic lines with a root separation ratio of 3:1 screened by 0.1% hygromycin were further studied. The homozygous lines of the T3 generation were used for phenotyping, and the expression levels of the target gene in the transgenic lines were identified by qRT-PCR.

The transgenic lines were planted in the paddy field in the experimental station of the Institute of Crop Sciences in Beijing, China (39°48′N; 116°28′E). The rice seeds were sown in a plastic box in late May or early June. Fifteen days after germination, the seedlings were transplanted in the paddy field. The agronomic traits, including plant height, tiller number, primary panicle length, branch number per panicle, and grain number per panicle were measured at maturity. The grain traits, including TGW, grain area, grain circumference, grain length, and grain width were measured by a precise grain phenotyping system (SC-G, http://www.wseen.com/). The data assays were performed by Duncan's Multiple Range test using SPSS version 16.0 software (IBM Corp., Armonk, NY, USA).

### Sequence Polymorphism Assays and Functional Marker Development

To identify the chromosomal location of the target gene, the cDNA sequence of *TaWRKY51* was used as a query to do BLAST (https://urgi.versailles.inra.fr/blast/blast.php), and the genomic sequences of Chinese Spring were obtained. The genome-specific primers which could specifically amplify the three members in subgenomes A, B, and D were designed accordingly. The primers are listed in Table 1. Wheat DNA was extracted from the young leaves with the cetyltrimethylammonium bromide method (Stewart and Via, 1993). TransStart Fast *Pfu* DNA Polymerase (TransGen Biotech, Beijing, China) was used for PCR amplification. The PCR was performed in a volume of 20 μl having 4 μl 5 × *Pfu* buffer, 0.4 μl fast *Pfu*, 3.2 μl dNTPs (25 mM), 0.8/0.8 μl forward and reverse primer (10 μM), 2 μl DNA (100 ng μl^−1^), and 8.8 μl ddH_2_O. The PCR conditions were 94°C for 5 min; 35 cycles of 94°C for 45 s, 60°C for 30 s, 72°C for 1 min; followed by a final extension of 72°C for 5 min. The PCR products were checked on agarose gel (1.2%), and the target bands were purified by V-Elute Gel Mini Purification Kit (ZomanBio, Beijing, China) and cloned into *pEASY*-Blunt vector for sequencing (https://www.transgen.com.cn/). Five clones for each accession were picked for sequencing by DNA Analyzer 3730XL (Applied Biosystems, USA). To get the full-length sequence of the target gene, M13F, M13R, and walking primers were used for sequencing (Table 1). The sequence of each clone was assembled using the Seqman program in the DNAStar software package (DNASTAR, Inc., WI, USA), and the consensus sequence of each accession was obtained in the same way. The genomic origin of each fragment was reconfirmed by comparing it with the reference genomic sequence (https://urgi.versailles.inra.fr/blast/blast.php). For functional marker development, the primers flanking the selected variation sites were designed, and agarose gels with proper intensity were used to visualize the differences.

### Morphological Characterization of Transgenic Rice

The three pure transgenic rice lines of T3 generation with various expression levels were selected for phenotyping. The root traits for transgenic rice were measured at different stages and in various environments. For 7-day-old seedlings, normally matured rice seeds were soaked in tap water for 3 days, and the germinated seeds were planted in a root phenotyping pouch (www.phytotc.com) filled with 20 ml ddH_2_O, PEG-6000 (−0.5 MPa), and 170 mM NaCl, respectively, and cultured in a growth chamber (25°C, 16 h light/30°C, 8 h dark cycle) for 7 days. Five seedlings were cultured in one pouch, and three replications were set for each line. The root traits were captured by a scanner (Epson expression 10000XL, Epson Corporation, Japan) and analyzed with WinRHIZO software (WinRHIZO Pro 2007). The root traits, including crown root number (CRN), maximum root length, total root length, total project area, total surface area, and root tip number (RTN) were measured. To examine the root growth of transgenic rice in the soil, 1-week-old seedlings were planted in the plastic tanks filled with paddy soil (length × width × height = 80 × 35 × 30 cm). The three replicates were set (10 plants per replicate). Ten plants with uniform size for each line were selected to measure the maximum root length and RN after a 6-week culture in the greenhouse. To further investigate whether *TaWRKY51* played roles in controlling RN, the rice roots of mature plants grown in the paddy field were collected postharvest, and RN was calculated after rinsing with tap water.

### Association Analysis

A software TASSEL (v 5.0) was used to identify the significant associations between agronomic traits and haplotypes for the NP1. The associations were considered statistically significant at *P* < 0.05. The effects of haplotype on each trait were also analyzed by an independent *t*-test at *P* < 0.05 (even 0.01) by SPSS (v 16.0).

## Results

### TaWRKY51s Are Evolutionarily Conserved in Triticeae

**Figure 1:**
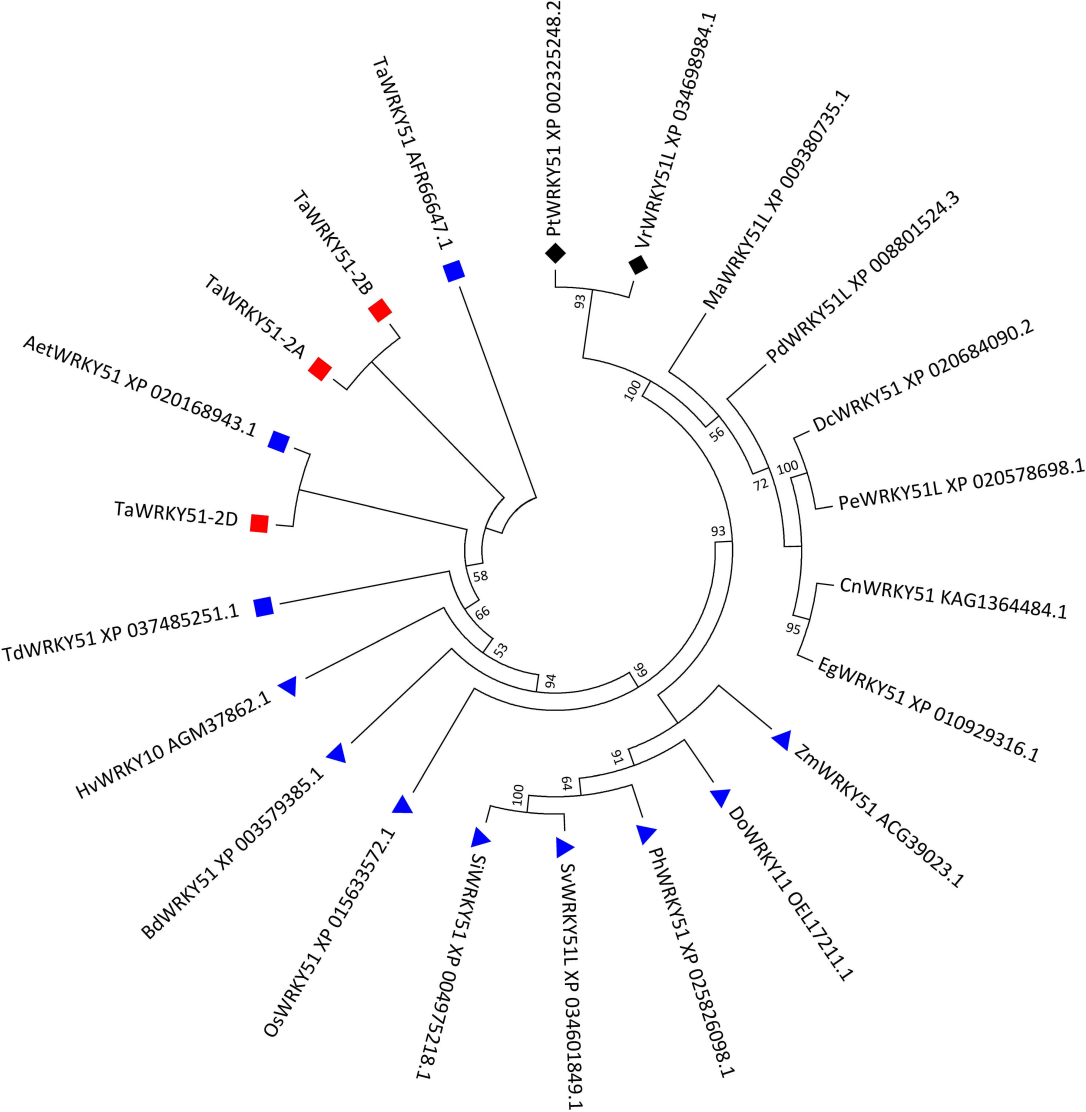
Phylogenetic tree of TaWRKY51s and the orthologs in the selected plant species. The evolutionary history was inferred by using the maximum likelihood method based on Poisson's correction model. The bootstrap consensus tree inferred from 1,000 replicates was taken to represent the evolutionary history of the taxa analyzed. The evolutionary analyses were conducted in MEGA7. Blue squares indicate wheat or wheat relatives. Blue triangles indicate the graminaceous crops or species. Black diamonds indicate the dicot species. Aet, *Aegilops tauschii*; Bd, *Brachypodium distachyon*; Cn, *Cocos nucifera*; Dc, *Dendrobium catenatum*; Do, *Dichanthelium oligosanthes*; Eg, *Elaeis guineensis*; Hv, *Hordeum vulgare*; Ma, *Musa acuminata*; Pd, *Phoenix dactylifera*; Os, *Oryza sativa*; Pe, *Phalaenopsis equestris*; Ph, *Panicum hallii*; Pm, *Panicum miliaceum*; Pt, *Populus trichocarpa*; Si, *Setaria italica*; Sv, *Setaria viridis*; Ta, *T. aestivum*; Td, *T. dicoccoides*; Vr, *Vitis riparia*; and Zm, *Zea maize*.

Three types of cDNA sequences of the target gene were obtained by PCR. They were named *TaWRKY51-2A, -2B*, and *-2D* according to their chromosome origins. The multi-alignments in the amino acid (AA) sequence indicated that the putative peptides of TaWRKY51-2A and -2B were completely identical, and TaWRKY51-2D has 98.7% identity with the other two members, differing in four AA resides. Since the AA variations in TaWRKY51-2D were far away from the WRKY domain, it might not cause any functional alteration (Supplementary Figure 1). A phylogenetic tree revealed that TaWRKY51s and the orthologs in Triticeae were first classified in the same clade, including goat grass, durum wheat, and barley, and then followed by the members of other gramineous species, including Brachypodium, rice, millet, and maize. The protein sequence identities between the members in Triticeae were higher than 96%, while the similarities between TaWRKY51s and other gramineous members were relatively lower (71–85%). Furthermore, the members of monocot and dicot species were split into different clades (Figure 1).

### Dynamic Expressions of *TaWRKY51s* in Various Wheat Tissues and Responses to Phytohormones and Abiotic Stresses

To investigate the potential involvements of *TaWRKY51s* in various biological processes, the upstream promoter sequences (about 3 kb) obtained from the reference genome of Chinese spring were used to identify *cis-acting* regulatory elements (http://bioinformatics.psb.ugent.be/webtools/plantcare/html/). A variety of *cis-acting* elements with different functions were identified, including hormone and stress responsive elements, and the elements involved in plant growth and development. The hormone responsive elements mainly contained ABA response elements (ABREs), MeJA responsive elements (CGTCA/TGACG-motif), gibberellin-responsive element (P-box), and salicylic acid responsive elements (TCA-element). The stress responsive elements included drought responsive elements (DRE core and MBS), low temperature responsive elements (LTR), wound responsive elements (WUN-motif and WRE3), and stress responsive elements (STRE). The elements regulating the plant growth and development contained meristem, endosperm expression elements, and zein metabolism regulation elements (Supplementary Figure 2). Comparatively, the number and types of *cis-acting* elements in *Pro-2D* were markedly higher than those of *Pro-2A* and *Pro-2B*, which might result in the differential transcriptions (Supplementary Table 1).

**Figure 2:**
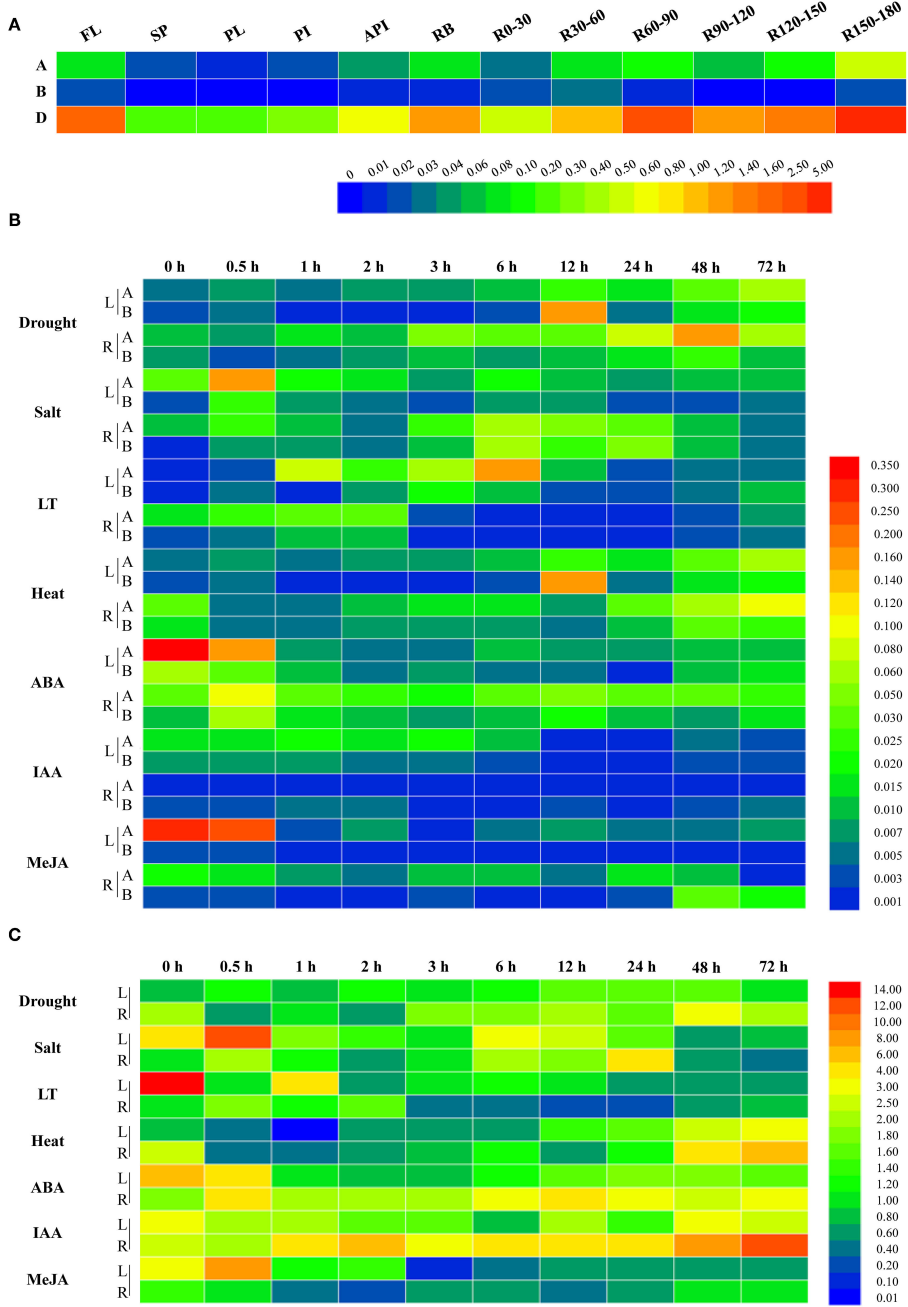
Expression patterns for *TaWRKY51s* in different tissues and under various abiotic stress and phytohormone conditions. **(A)** The tissue specific expressions of *TaWRKY51s* at the flowering stage in wheat. The relative expression levels of *TaWRKY51-2A* and *-2B* **(B)** and *TaWRKY51-2D* **(C)** in the wheat leaves and roots under various abiotic stress conditions and upon different phytohormones. A *TaWRKY51-2A*; B *TaWRKY51-2B*; API, antepenultimate internode; FL, flag leaf; L, leaf; LT, low temperature; PI, penultimate internode; PL, peduncle length; SP, spike; R, root; Rx-y, indicates the root section from x to y centimeters.

To further uncover the involvement of target genes in wheat growth and development, the qRT-PCR was performed and the results showed that the expression levels for the three members differed substantially at the flowering stage. As shown in Figure 2A, the expressions of *TaWRKY51s* in roots, root bases, and flag leaves were relatively higher than those in spikes and stems, including peduncles, penultimate internodes, and antepenultimate internodes. Comparatively, *TaWRKY51-2D* maintained the highest transcriptions in various tissues, then followed by *TaWRKY51-2A*, and the lowest transcriptions occurred in *TaWRKY51-2B*. For *TaWRKY51-5A*, the maximum expression was observed in deep roots (at 150-180 cm), then followed by R60-90, R120-150, flag leaves, and root bases. For *TaWRKY51-2B*, relatively higher expressions occurred in R30-60, R150-180, R0-30, and flag leaves (Figure 2A). The maximum expression of *TaWRKY51-2D* was identified in R150-180, then followed by R60-90, flag leaves, R120-150, and root bases (Figure 2A).

To further investigate their responses to phytohormones and environmental stimuli, two-leaf wheat seedlings were subjected to various treatments, and the dynamic expression patterns were observed (Figures 2B,C). Overall, the *TaWRKY51s* were induced by various abiotic stresses, but the basal transcriptions for the three members differed remarkably. The highest transcriptions were observed in *TaWRKY51-2D*, then followed by *TaWRKY51-2A* and *-2B* (Figures 2B,C). *TaWRKY51-2A* was significantly induced by drought, salt, low temperature, and heat stress in both the shoots and roots, while the expression patterns and peak transcriptions varied markedly under different stresses (Figure 2B). For phytohormones, *TaWRKY51-2A* was slightly induced by ABA in the roots but suppressed in the shoots, and was inhibited by IAA and MeJA in both the leaves and roots (Figure 2B). *TaWRKY51-2B* maintained very low expression levels all the time. It was induced by drought, salt, cold, and heat stress, repressed by ABA, and showed insensitive to MeJA and IAA. Comparing with the expression levels of the other two members, its expression can be neglected, suggesting that it might not be a major effect gene (Figure 2B). Comparing with the low transcriptions of *TaWRKY51-2A* and *-2B*, the transcripts of *TaWRKY51-2D* were extremely high regardless of with or without stress/treatment (Figure 2C). It was induced by drought, salt, and heat in both the shoots and roots, inhibited by cold in the leaves, and transiently induced by cold, ABA, and IAA in roots, by MeJA in the leaves (Figure 2C).

### Subcellular Localization of TaWRKY51

**Figure 3:**
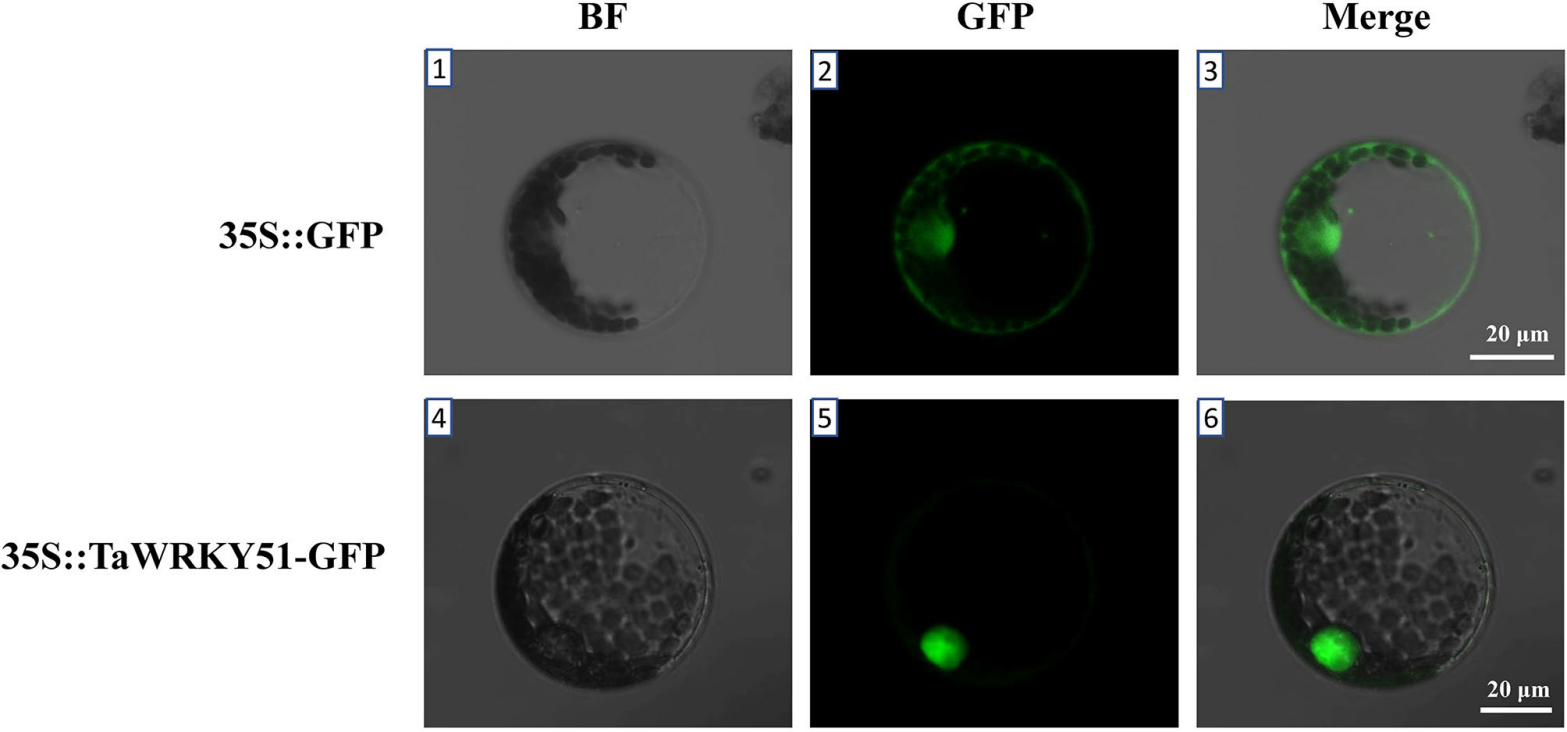
TaWRKY51 specifically locates in the nucleus of the wheat protoplast. The constructs 35S::TaWRKY51-green fluorescent protein (GFP) and 35S::GFP were transformed into wheat protoplast by polyethylene glycol (PEG)-mediated method respectively. 1–3, wheat protoplast expressing GFP as a control. 4–6, wheat protoplast expressing TaWRKY51-GFP fusion protein. The pictures were taken in bright and black fields 36 h after transformation by a laser scanning confocal microscope.

The subcellular localization of TaWRKY51 was first predicted online (https://predictprotein.org/), and the result showed that it was localized in the nucleus (Supplementary Figure 3). To further test the result, a recombinant construct of pJIT-163-35S::*TaWRKY51*-*GFP* was transformed into wheat protoplast, and the pJIT-163-35S::*GFP* vector was used as the control. The green fluorescence signal for GFP control was observed in the cell nucleus and cytoplasm, while TaWRKY51-GFP fusion protein was only observed in the nucleus (Figure 3), suggesting that TaWRKY51 protein was specifically localized in the cell nucleus, consistent with its role as a transcription factor.

### Nucleotide Sequence Polymorphism Assays and Molecular Marker Development

**Figure 4:**
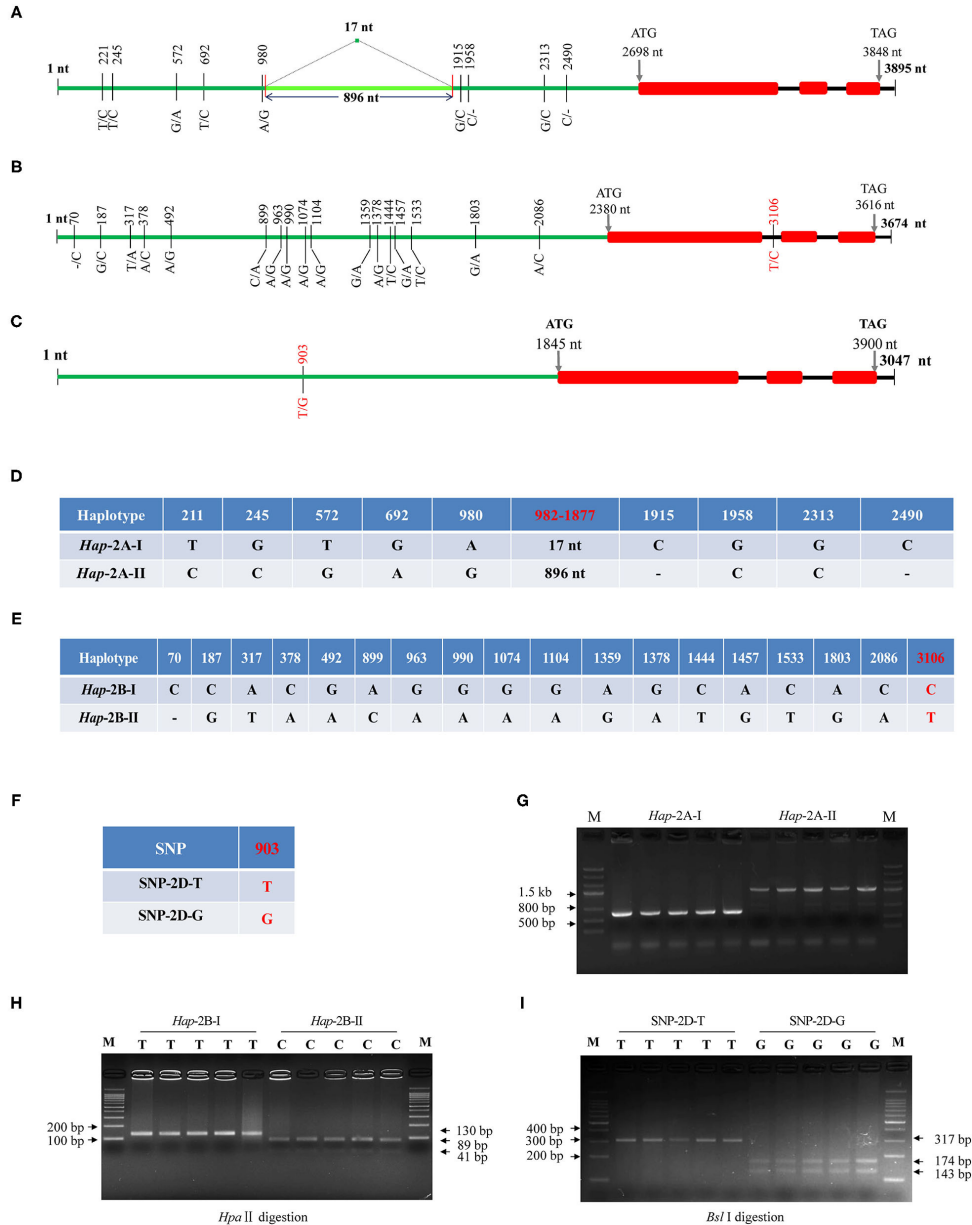
Gene structures and DNA polymorphisms of *TaWRKY51s*. Gene structures and variation distributions of *TaWRKY51-2A* **(A)**, *-2B* **(B)**, and *-2D* **(C)**. The compositions of the haplotypes/alleles for *TaWRKY51-2A* **(D)**, *-2B* **(E)**, and *-2D* **(F)**; functional marker for *TaWRKY51-2A* **(G)**, *-2B* **(H)**, and *-2D* **(I)**. The red numbers indicate the position where the functional markers were designed. The black lines indicate introns, red blocks indicate the exons of *TaWRKY51s*, green lines represent the upstream promoters. The size of blocks and lines is proportional to the length of the DNA sequence.

The obtained cDNA sequences were used as a query to do BLAST (https://urgi.versailles.inra.fr/blast/). Three scaffolds were obtained, i.e., scaffold_096602_2AL, 131202_2BL, and 160554_2DL, and the genome-specific primers were designed at DNA polymorphic sites accordingly (Table 1). Gene structure assays showed that *TaWRKY51s* contained three exons and two introns, and their structures were evolutionarily conserved. The genomic DNA of the three members, covering the upstream promoter and gene coding regions were isolated, and the fragment sizes were 2999/3895, 3674, and 3407 bp, respectively. Sequencing results showed there were nine variations in *TaWRKY51-2A*, and all the variations were located in the promoter regions. Among which, six were single nucleotide polymorphisms (SNPs), two single nucleotide insertion/deletions (InDels), and one structure variation, a 17-nt fragment was replaced by a fragment of 896 nt (locating between 982 and 1,877 nt) (Figures 4A,D). As shown in Figure 4C, the variations were genetically linked. As the consequence, two haplotypes, i.e., *Hap-*2A-I and *Hap-*2A-II, were formed in the polymorphism assay panel (PAP) (Figure 4D). Based on the structure variation, an A-genome-specific molecular marker was developed, which could effectively identify the two haplotypes in the natural populations (Figure 4G).

Eighteen SNPs were identified in *TaWRKY51-2B*. Among them, 17 SNPs were located in the promoter regions, and one in the first intron (Figures 4B,E). As shown in Figure 4E, all the variations were genetically linked, thus only two haplotypes were formed in the PAP. Based on the SNP at 3,106 nt (T/C), a cleaved amplified polymorphic sequence (CAPS) marker was developed. When the nucleotide was T (*Hap-*2B-I), the target fragment could not be digested and a single band with 130 nt presented on the gel; if it was C (*Hap-*2B-II), the PCR fragment could be cut by *Hpa*II and resulted in two bands on agarose gel with the sizes of 41 and 89 nt (Figure 4H).

For *TaWRKY51-2D*, a single SNP at 903 nt (T/G) was identified in the promoter region, while no variation was identified in the gene encoding regions. Based on the SNP, a CAPS marker was developed. When the nucleotide at 903 nt was T (SNP-2D-T), the PCR fragment could not be digested by *Sbl* I, and there was a 317-bp band on the agarose gel. If the nucleotide was G (SNP-2D-G), the PCR fragment could be cut by *Sbl* I and resulted in two bands on agarose gel with the sizes of 174 and 143 nt (Figure 4I).

### The Variations of *TaWRKY51s* Were Associated With Spike Size and Root Traits

**Figure 5:**
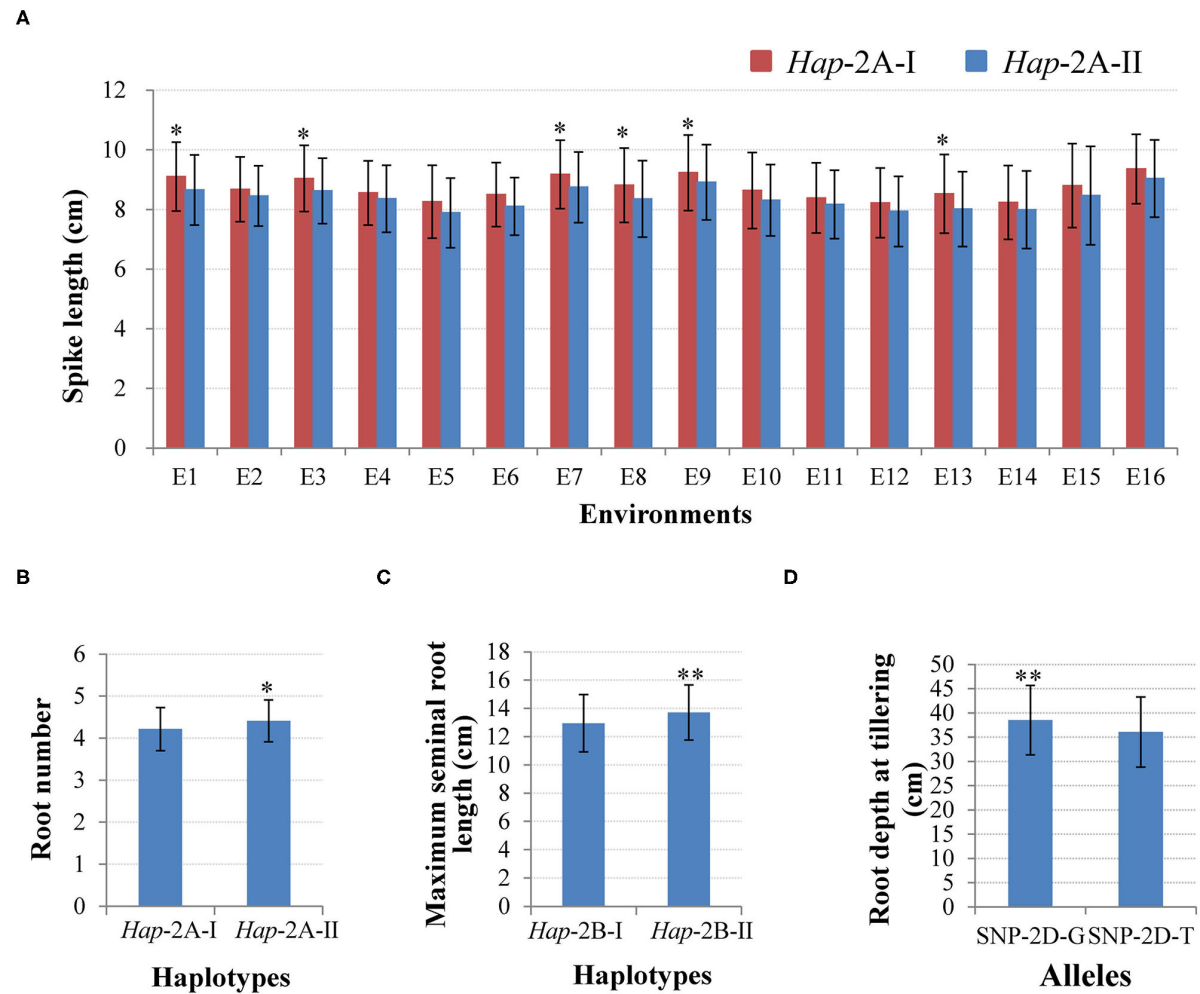
Phenotypic comparisons between different haplotypes/alleles for *TaWRKY51s*. Comparisons of spike length **(A)** and root number (RN) **(B)** for the two haplotypes of *TaWRKY51-2A*, maximum seminal root length for *TaWRKY51-2B* **(C)**, and root depth for the two alleles of *TaWRKY51-2D* at the tillering stage **(D)**. *, ** indicate significance at 0.05 and 0.01.

Two haplotypes of *TaWRKY51-2A* were identified in the NP1, i.e., *Hap*-2A-I and *Hap*-2A-II. *Hap*-2A-I was the most frequent haplotype with a frequency of 86.4%, then followed by *Hap*-2A-II (13.6%). The phenotyping results showed that the spike lengths of *Hap*-2A-I accessions were larger than those of *Hap*-2A-II, and the differences reached a significant level (*P* < 0.05) under 6 of 16 environments (Figure 5A). Therefore, *Hap*-2A-I was regarded as an elite haplotype for a large spike in wheat. Furthermore, *TaWRKY51-2A* was associated with the RN at the seedling stage, and *Hap*-2A-II containing accessions had substantially more roots than the genotype of *Hap*-2A-I (*P* < 0.05) (Figure 5B).

For *TaWRKY51-2B*, there were also two haplotypes present in the NP1. Among them, *Hap*-2B-II was the predominant haplotype, accounting for a rate of 78.8%, and the frequency for *Hap*-2B-I was 21.2%. The phenotypic comparisons showed that the spike lengths of *Hap*-2B-I accessions were longer than those of *Hap*-2B-II under all the environments, however, the difference only reached a significant level in 1 of 16 environments (*P* < 0.05), suggesting its effect in regulating spike size might be marginal (Supplementary Figure 4). Further *t*-test results revealed that the maximum root length of *Hap*-2B-II accessions was significantly longer than that of *Hap*-2B-I (Figure 5C).

There were two alleles identified in *TaWRKY51-5D* in the PAP and the natural populations. Among the two alleles, the allele-G was the predominant one in both the landrace and modern variety populations. Student's *t*-test results revealed that the root depth of allele-G accessions was significantly deeper than that of allele-T accessions at the tillering stage in wheat (Figure 5D).

### Geographic and Temporal Distributions of the Haplotypes/Alleles for *TaWRKY51s* in China

**Figure 6:**
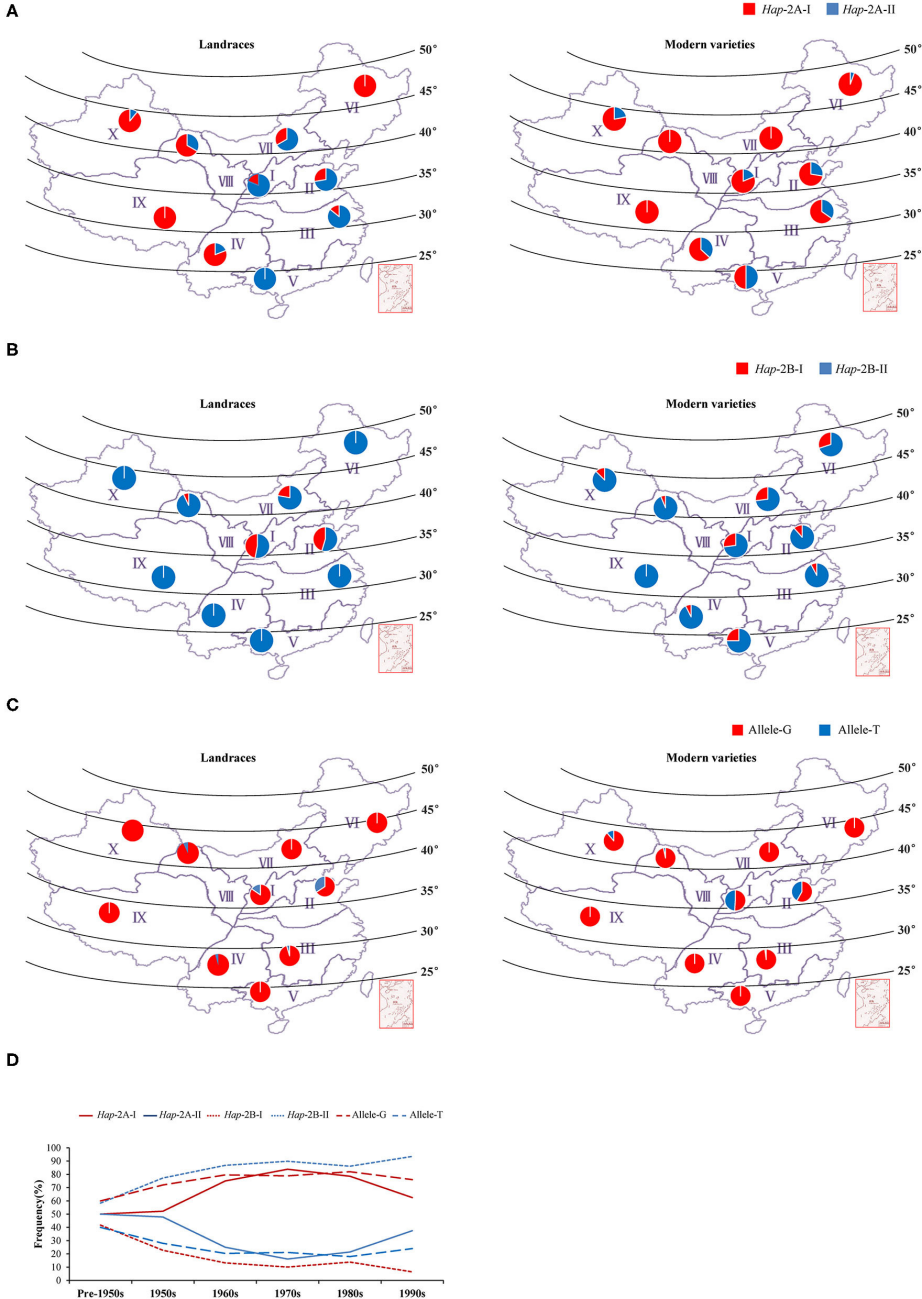
Geographic and temporal distributions of the haplotypes/alleles for *TaWRKY51s* in the Chinese landraces and modern cultivars in the 10 wheat zones. Geographic distributions of the haplotypes for *TaWRKY51-2A* **(A)**, *-2B* **(B)**, and *-2D* **(C)** in the Chinese landrace and modern variety populations. The frequency alterations for the haplotypes/alleles of *TaWRKY51s* in modern varieties were released in different time periods in China **(D)**.

The wheat production area in China was divided into 10 major agro-ecological zones based on the geographical location, wheat type, growing season, and various responses to temperature and photoperiod (Liu et al., 2017), among which Zones I–IV, and VI covers 85% of the area and production (He et al., 2001). To determine whether the elite haplotypes of *TaWRKY51s* were selected in the process of wheat breeding, their geographic distributions were investigated in the two natural populations. In the NP2 (landraces), *Hap*-2A-II was the dominant haplotype in the main wheat-producing area, including Zone I, II, III, V, and VII, and the frequencies were 81, 72, 86, 100, and 67%, respectively, whereas *Hap*-2A-I was the principal haplotype in the rest zones (Figure 6A). In the NP3 (modern varieties), the rate of *Hap*-2A-I increased remarkably in Zone I, II, III, V, and VII, and the frequencies were 81, 72, 65, 50, and 100%. From the Chinese landraces to modern cultivars, the frequencies of the *Hap*-2A-I increased in Zone I (19–81%), II (28–72%), III (14–65%), V (0–50%), and VII (34–100%) (Figure 6A), indicating that the large spike haplotype was strongly selected in the Chinese wheat breading. To assess the changes in haplotype frequency over the time course, the Chinese modern varieties were divided into six subgroups according to the released times. As shown in Figure 6D, the frequency for *Hap*-2A-I increased first and then decreased gradually, whereas the overall trend was increasing.

For *TaWRKY51-2B, Hap*-2B-II was the predominant haplotype in both NP2 and NP3 (Figure 6B). In the NP2, *Hap*-2B-I was only identified in a few wheat zones, including Zone I (47%), II (45%), VII (22%), and VIII (7%). In the NP3, an increasing trend for *Hap*-2B-I was observed in most of the wheat zones. It was identified in 9 of 10 wheat zones, except Zone IX, but the proportions were much lower than that of *Hap*-2B-II. Intriguingly, the frequencies of *Hap*-2B-I in two major wheat zones were substantially reduced, i.e., Zone I (47–27%) and II (45–12%) (Figure 6B). *Hap*-2B-II was maintained as a predominant haplotype all the time, and its frequency continuously increased over time (Figure 6D).

The two alleles of *TaWRKY51-2D* were identified in the natural populations, and allele-G was the predominant haplotype in both NP2 and NP3 (Figure 6C). In the NP2, allele-T was only identified in 5 of 10 zones, and its proportions in these zones were much lower relative to allele-G, i.e., Zone I (15%), II (34%), III (5%), IV (5%), and VIII (7%). In the NP3, the overall trend of allele-T was not changed in most zones, except in Zone I (49%) and II (42%). The proportion increase of shallow root depth allele (allele-T) in modern varieties in major zones suggests that the deep root allele (allele-G) was not positively selected in the wheat breeding (Figure 6C). However, the predominant allele-G maintained a higher frequency over time (Figure 6D).

### Overexpression of *TaWRKY51* Resulted in More Crown and Lateral Roots in Rice

**Figure 7:**
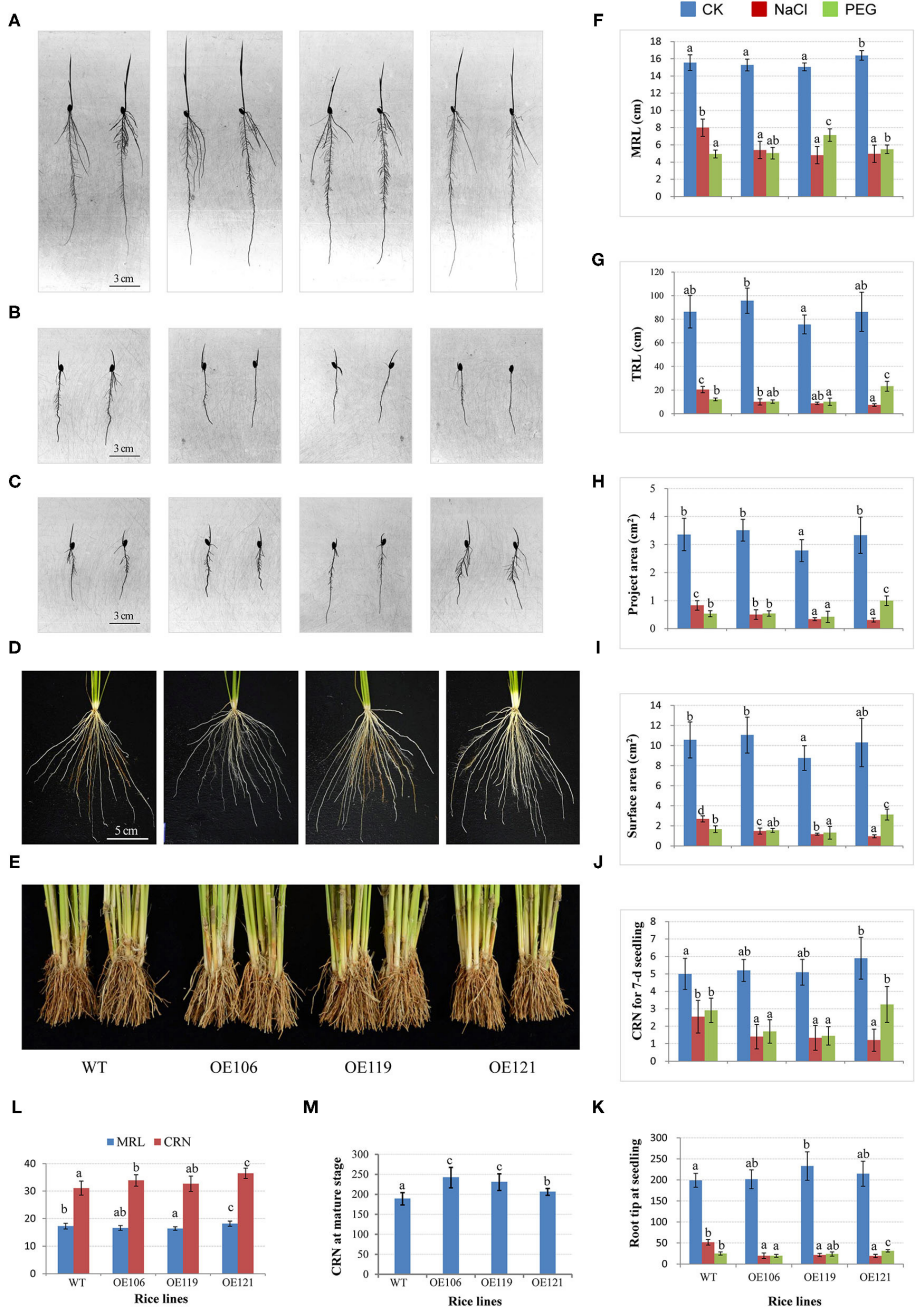
Comparisons of root traits between *TaWRKY51-overexpressing* rice and the wild-type (WT) control at various developmental stages. The root images of 7-day-old seedlings cultured in pouches under normal **(A)**, NaCl **(B)**, and PEG **(C)** stressed conditions. The root images for 6-week-old seedlings **(D)** and mature plants **(E)** were cultured in the paddy soil. Comparisons of maximum root length **(F)**, total root length **(G)**, total project root area **(H)**, total surface area **(I)**, crown root number (CRN) **(J)**, and root tip number (RTN) **(K)** for rice seedlings grown in the root pouches **(L)**, comparisons of maximum root length and CRN for seedlings cultured in the paddy soil. **(M)** Comparison of CRN for mature plants grown in the paddy field.

To further investigate the function of the target gene, *TaWRKY51-2A* was transferred into rice by an agrobacteria-mediated method, and the morphological traits of the resulting plants were extensively observed. Three homozygous lines with various expression levels of *TaWRKY51* were selected for the trait measurements, the highest expression was identified in OE119, then followed by OE121 and OE106 (Supplementary Figure 5). The root traits for 7-day-old rice seedlings cultured in the root pouches were first measured. There were no consistent differences in the maximum root length, total root length, total project area, and total surface area between WT and transgenic lines OE106 and OE121 under normal growing conditions, while the values for OE119 were significantly smaller than WT in these traits (Figures 7A–C,F–I). However, the CRN and RTN of all transgenic lines were higher than WT, and the differences reached significant levels in OE119 and OE121, respectively (Figures 7A–C,J–K). Under salt-stressed conditions, all the measured root traits were dramatically reduced in both WT and transgenic plants, however, the reductions in transgenic lines were much more significant than that of WT, indicating that the overexpression of *TaWRKY51* increased the sensitivity of transgenic rice to high salinity (Figures 7A–C,F–L). Under PEG-stressed conditions, the differences between transgenic plants and WT were not so evident in maximum root length, total root length, the total projected root area, total surface area, and RTN, the values for these traits were significantly larger in OE121, no difference in OE106, and significantly lower in OE119, whereas the maximum root length of OE119 and OE121 were substantially longer than WT, suggesting that the inhibiting effects of water deficit on the transgenics and the WT control were similar (Figures 7A–C,F–K).

For seedlings cultured in the paddy soil, the maximum root length of OE121 was significantly higher, while the values for OE106 and OE119 were lower than that of WT. However, the CRN for all the transgenic lines was higher than WT, and two of the three lines reached significant levels (Figures 7D,L). Moreover, the CRN of mature plants was also substantially higher than the WT control (Figures 7E,M).

### Transgenic Rice Overexpressing *TaWRKY51* Had Improved Yield-Contributing Traits

**Figure 8:**
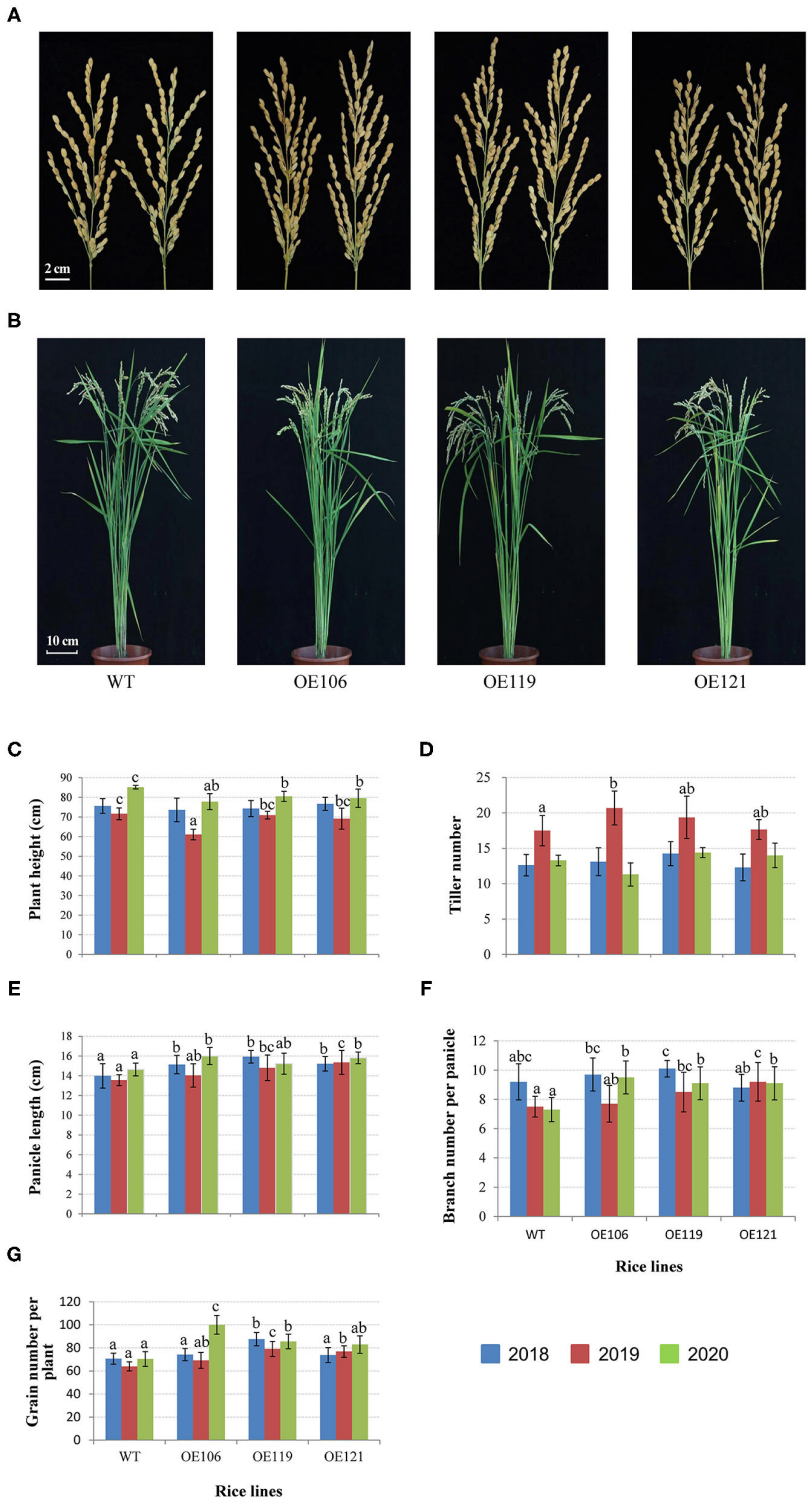
Comparisons of agronomic traits between *TaWRKY51* transgenic lines and the WT control. The images of rice panicles **(A)** and mature plants **(B)**. Comparisons of plant height **(C)**, tiller number **(D)**, panicle length **(E)**, panicle branch number **(F)**, and grain number per plant **(G)**. The images of rice panicles and plants were photographed in 2019. The agronomic traits were measured at the experimental station of ICS-CAAS during 2018–2020.

The agronomic and grain traits of the transgenic rice were continuously measured for 3 years. As shown in Figure 8, the plant height of the transgenic lines was remarkably reduced in 2019 and 2020, while no significant difference was identified in 2018 (Figures 8B,C). The tiller numbers of transgenic plants were higher than the WT control in 2019, and the difference in OE106 reached a significant level, yet no significant difference was observed in 2018 and 2020 (Figure 8D). The primary panicle lengths of all the transgenic lines were significantly longer than WT in 3 years (Figures 8A,E). Furthermore, the transgenic plants had more panicle branches in most cases, and the differences were significant in 2019 and 2020 (Figures 8A,F). Accordingly, the grain number per plant of all the transgenic lines was higher than the WT control in three years, and significant differences were observed in most of the transgenic lines (Figures 8A,G).

### Overexpression of *TaWRKY51* Increased the Grain Length of Transgenic Rice

**Figure 9:**
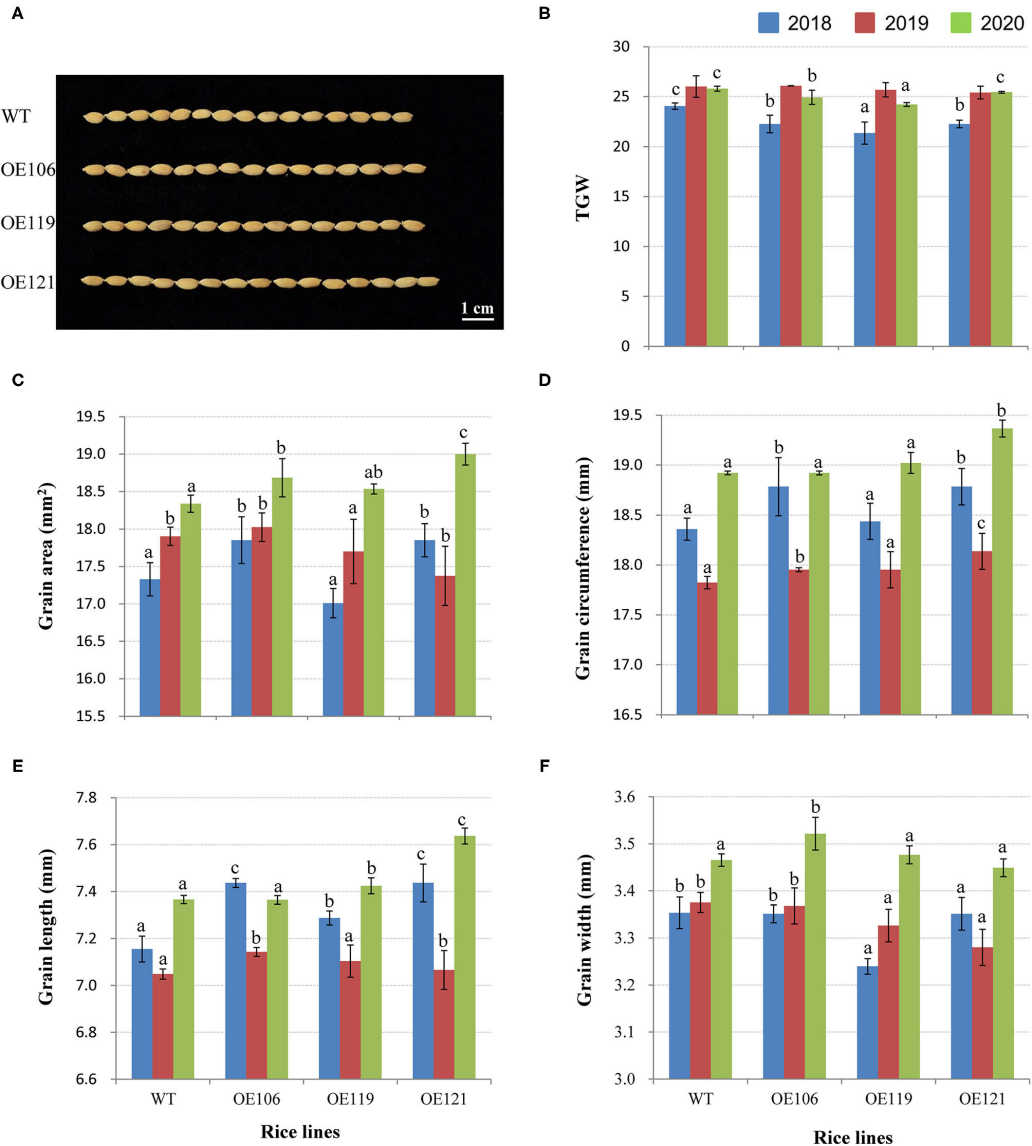
Comparisons of grain traits between the *TaWRKY51-overexpressing* rice and the WT control. Comparisons of grain length **(A,E)**, TGW **(B)**, grain area **(C)**, grain circumference **(D)**, and grain width **(F)** of transgenic rice and the WT control. The images of rice grains were photographed in 2019.

To discover the potential role of TaWRKY51 in the development of grain size, the grain traits of transgenic rice were measured, including TGW, grain area, grain circumference, grain length, and width. As shown in Figure 9A, the TGW of WT were significantly higher than the transgenic lines in 2018, while no difference was identified in 2019, and the values of non-transgenic plants were higher in most cases (2/3) in 2020. However, the values for grain area, circumference, and length of the transgenic plants were evidently greater than that of WT in most cases, and the differences reached significant levels in some cases (Figures 9B–E). The grain width of WT plants was larger than the two transgenic lines in 2018 and 2019, whereas no difference was observed in 2020 (Figure 9F).

## Discussion

The WRKY TFs are plant-specific regulators involved in a variety of biological processes (Grunewald et al., 2012; Cai et al., 2014; Tian et al., 2017) and responding to various abiotic stresses (Lee et al., 2018). In the present study, we isolated the three members of *TaWRKY51* in common wheat. Amino acid sequence assays showed that they were highly conserved in common wheat (Supplementary Figure 1). The phylogenetic tree showed that TaWRKY51s and their orthologs in monocots were classified in the same clade, while the members from dicots were clustered in the other one (Figure 2), suggesting that WRKY51 might have occurred after the segregation of monocots and dicots. Subcellular localization revealed that TaWRKY51 was specifically localized to the nucleus, which is consistent with previously reported TaWRKY10 (Wu et al., 2008; Wang et al., 2013).

The spatiotemporal expression is a direct indication of the involvement of a gene in biological processes. *Cis-acting* element assays showed the presence of multiple regulatory elements in the promoter regions of *TaWRKY51s*, including drought, ABA, and MeJA. Gene expression results further confirmed that the three members were implicated in the response to drought and ABA (Figures 2B,C). However, their responses to MeJA were not so evident despite the presence of several JA-responsive elements in the promoters, suggesting that other unknown inhibitors might be implicated in regulating their transcriptions. Comparing the expression patterns of the three members at the flowering stage, it was evident that their transcripts in flag leaves, root bases, and roots were relatively higher than in the spikes and stems (Figure 2A), hinting that *TaWRKY51s* might be involved in the development of traits associated with the photosynthesis and root development (Hu et al., 2018).

Accumulated data have documented that WRKY TFs are multifunctional regulators in plant growth and development. Several WRKY TFs were identified as the regulators of root development. For instance, AtWRKY46 modulates the development of lateral roots under adverse conditions (Ding et al., 2015). AtWRKY75 is involved in regulating the development of root architecture (Devaiah et al., 2007). CbNN1 positively controls the formation of adventitious roots (Wang et al., 2019). TaWRKY51 promotes the formation of lateral roots in wheat (Hu et al., 2018). In the current research, our association results showed that *Hap*-2A-II and *Hap*-2B-II were associated with more seminal RN and longer roots at the seedling stage (7 days after germination), respectively, and allele-G was associated with root depth at the tillering stage, suggesting that *TaWRKY51s* might have a role in regulating the development of root architecture in wheat.

The transgenic experiments showed the RTN for transgenic rice seedlings were more than that of WT under normal growth conditions (cultured in root pouches) (Figures 7A,J), indicating that the transgenic plants had more lateral root, which is consistent with a previous report (Hu et al., 2018). Further experiments in the paddy soil revealed that the CRNs of the transgenic rice lines were significantly higher than that of WT at both the seedling and mature stages, strongly demonstrating that the heteroexpression of *TaWRKY51* can enhance CRN in rice. A previous study has shown that *TaWRKY51* is involved in regulating the development of lateral roots, while our data strongly supported that *TaWRKY51* has a role in controlling the RN and root length, the difference in these results might be attributed to the fact that the former research mainly focused on the root morphology at the seedling stage under hydroponic conditions (Hu et al., 2018).

In reality, the development of plant roots in soil is modulated by numerous environmental factors and roots are key organs for water and nutrients uptake. The increase of CRN and lateral roots will inevitably contribute to the absorption of water and nutrients in the soil, which is conducive to the improvement of GY, especially underwater/nutrient-deficient conditions. GY is a complex trait determined by three major components, TGW, grain number per spike, and spike number/effective tiller number per unit area (SN/TN) (Cao et al., 2020). The enhancement of either component might be beneficial to enhance GY. A few reports have shown that WRKY TFs are involved in the regulation of agronomic traits associated with GY. For instance, *Loose panicle* 1 (*LP1*), encoding a Group I WRKY member, is involved in controlling panicle length and primary branch number in foxtail millet (Xiang et al., 2017). OsWRKY36 plays a negative role in regulating the plant height and grain size by directly binding to the promoter of the *SLR1* gene and protecting SLR1 from GA-mediated degradation in rice (Lan et al., 2020). In this study, our association results revealed that *Hap*-2A-I was associated with a larger spike in the 6 of 16 environments and strongly selected in the process of wheat breeding in China. The transgenic experiments showed that the panicle size of *TaWRKY51*-*overexpressing* the rice plants was significantly larger than the WT control (Figure 8E), demonstrating that the association results were reliable. Simultaneously, the branch number and grain number per panicle of transgenic rice were all significantly enhanced (Figures 8F,G). Furthermore, the grain traits including grain length, grain circumference, and grain area for the transgenic rice lines were also increased markedly (Figure 9), demonstrating that *TaWRKY51* plays a positive role in the development of GY contributing traits. Given that TaWRKY51 has positive roles in the development of root architecture and GY contributions traits, it has a broad application prospect to enhance yield potential in major crops.

Comparing the frequencies of different haplotypes of *TaWRKY51s* in the NPs, the frequency of *Hap*-2A-I, the elite haplotype for a long spike, was significantly increased from the Chinese landraces to modern varieties, revealing that it was positively selected in wheat breeding (Figure 6A), and this hypothesis is consistent with the history of GY-oriented breeding. Simultaneously, the frequency of *Hap*-2A-II, the haplotype for more seminal root was dramatically reduced from the landraces to modern cultivars. For *TaWRKY51-2B*, its geographic distributions increased in most wheat zones, except the two major wheat zones I and II. However, the temporal frequencies of *Hap*-2B-I declined over the time course, suggesting that the imposed selection on *Hap*-2B-I was unconscious and it might be a marginal gene in controlling the spike length (Figures 6B,D). This speculation was supported by the gene expression patterns of *TaWRKY51-2B* (Figures 2A,B). The transcription levels of *TaWRKY51-2B* were substantially lower relative to *TaWRKY51-2A* and *-2D* in all the observed wheat tissues and under various environmental conditions (Figures 2A–C). For *TaWRKY51-2D*, the proportions of deep root allele-G were maintained at a high level in both the landraces and released cultivars, however, its proportions in the two major wheat zones declined dramatically (Figures 6C,D), hinting that the elite allele for deep root was not selected in wheat breeding. According to our data, we postulate that the roles of TaWRKY51s in regulating the root architecture development have not received enough attention in GY-oriented breeding (Figures 6A–C), which might be a potential reason for the limitations under abiotic stress in modern wheat varieties (Li et al., 2019). To mitigate climate change and enhance tolerance to abiotic stress, the elite haplotype/allele for root traits might be a potential option for root architecture improvement in wheat.

Marker assisted selection is one of the important tools for precise breeding but has not been fully exploited in wheat breeding because of the limited availability of useful markers and the complexity of agronomic traits, especially multi-gene-controlled quantitative traits. The functional markers are developed based on the allelic variants of a functional gene, which can accurately discriminate the alleles of targeted genes, and are ideal molecular markers for MAS in wheat breeding (Liu et al., 2012). Our results clearly showed that *TaWRKY51s* have positive contributions to the development of root architecture and GY related traits, the developed functional markers are effective in identifying different alleles/haplotypes in natural populations and can be applied in wheat breeding.

## Conclusion

*TaWRKY51s* are multifaceted regulators involved in controlling the root architecture and the development of several agronomic traits related to GY. The favorable haplotype of *TaWRKY51-2A* for the large spike was positively selected in wheat improvements. However, their roles in the regulation of root architecture development have not received enough attention, and merit consideration in root trait improvement to mitigate climate change in the future.

## Data Availability Statement

The original contributions presented in the study are included in the article/Supplementary Materials, further inquiries can be directed to the corresponding authors.

## Author Contributions

XM, YL, and CL conceived and designed the experiments. YL, YZ, XC, CL, LY, JZ, JW, and LL performed the experiments. XM, YL, and YZ wrote the manuscript. MR, XM, CW, and RJ revised the manuscript.

## Funding

This work was supported by the National Natural Science Foundation of China (32061143040) and the China Agriculture Research System of MOF and MARA (CARS-03).

## Conflict of Interest

The authors declare that the research was conducted in the absence of any commercial or financial relationships that could be construed as a potential conflict of interest.

## Publisher's Note

All claims expressed in this article are solely those of the authors and do not necessarily represent those of their affiliated organizations, or those of the publisher, the editors and the reviewers. Any product that may be evaluated in this article, or claim that may be made by its manufacturer, is not guaranteed or endorsed by the publisher.
