# Supplementary material for: Transcription Factor TaWRKY51 Is a Positive Regulator in Root Architecture and Grain Yield Contributing Traits
Source: Front Plant Sci. 2021 Oct 21;12:734614. doi: 10.3389/fpls.2021.734614 (PMC8567066; doi:10.3389/fpls.2021.734614)
Supplement: Supplementary file 1 [file Data_Sheet_1.docx]

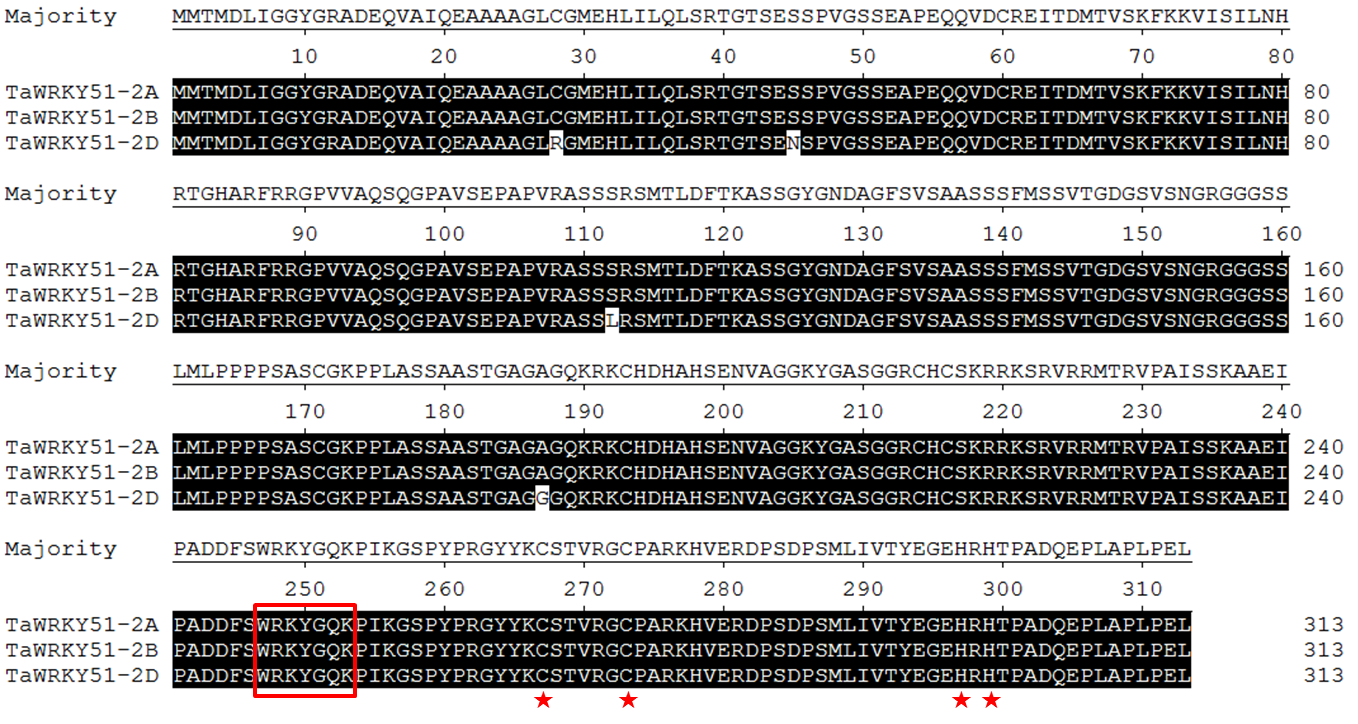


**Fig. S1 Comparisons of amino acid sequences of TaWRKY51s.**

Note, amino acid resides matching the consensus sequence exactly were shaded with solid black. Rectangle indicates the conserved WRKY motif and red stars indicate the conserved amino acid in the C_2_H_2_ Zinc finger motif of the WRKY domain.


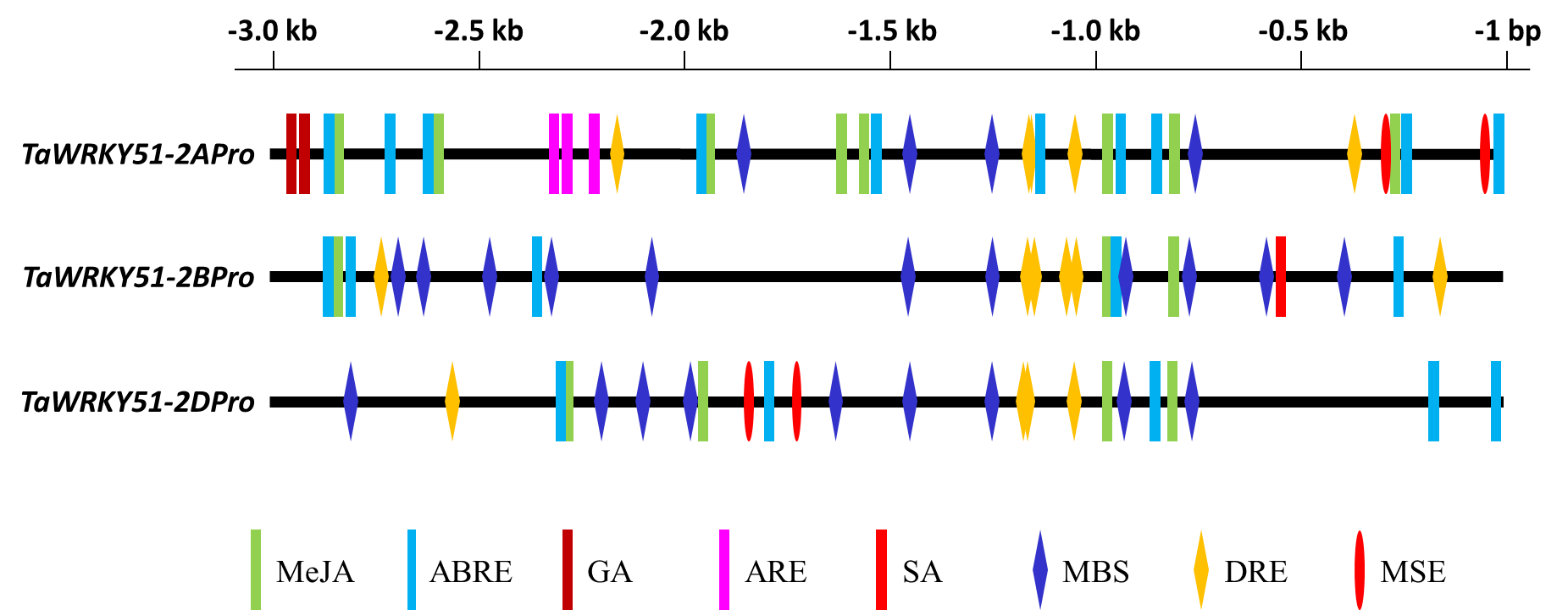


Fig S2. Distributions of various *cis-acting* regulatory elements in the promoter regions of *TaWRKY51s* in reference genome of Chinese spring.


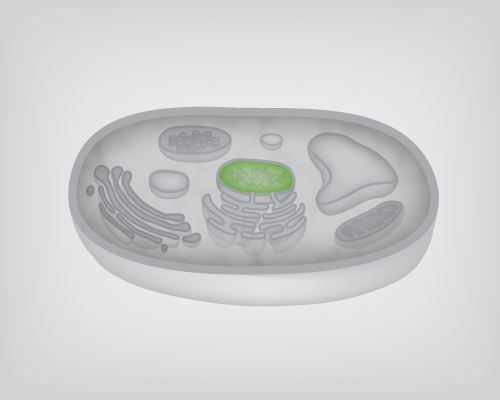


Fig. S3 TaWRKY51 was predicted localized in the nucleus with a confidence score of 38.


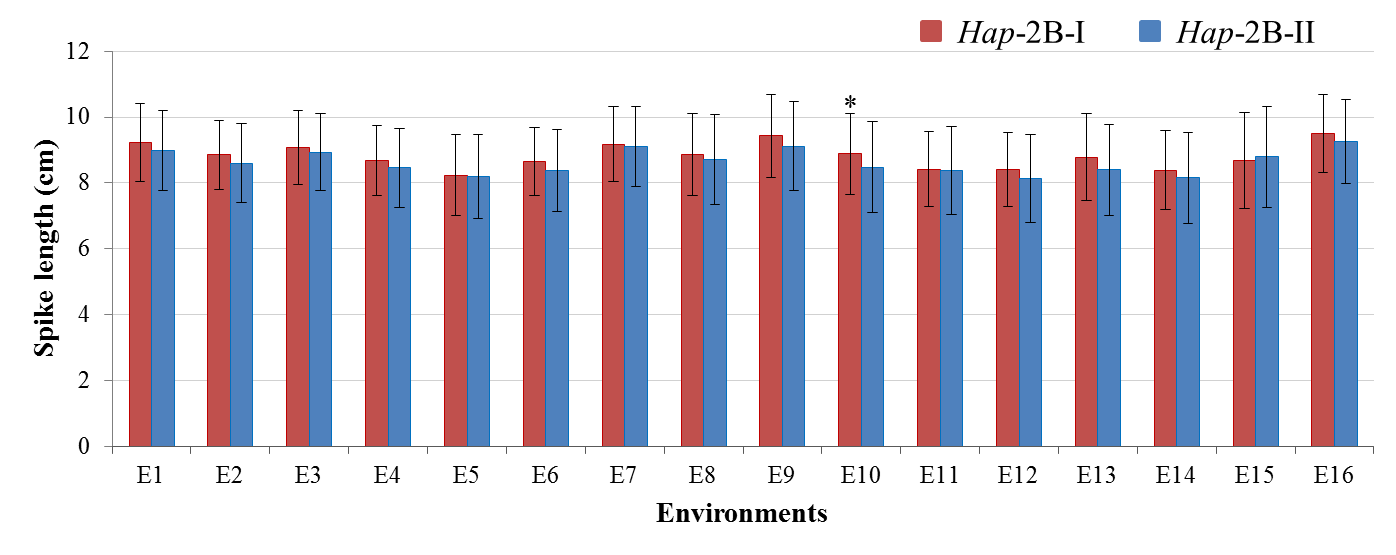


Fig. S4 Comparisons of spike length for the two haplotypes of *TaWRKY51-2B* in 16 environments.

Fig. S5 Relative expression levels of *TaWRKY51* in different transgenic rice lines

Table S1 Comparisons of *cis-acting* elements identified in the promoters of *TaWRKY51s*

| **Name** | **Number of *cis-acting* elements** | | | **Function** | **Type** |  |
| --- | --- | --- | --- | --- | --- | --- |
|  | **Pro-2A** | **Pro-2B** | **Pro-2D** |  |  |  |
| ABRE | 12 | 12 | 8 | *cis-acting* element involved in the abscisic acid responsiveness | phytohormone response | |
| CGTCA/TGACG-motif | 3 | 3 | 3 | *cis-acting* regulatory element involved in the MeJA-responsiveness |  | |
| P-box | - | - | 1 | gibberellin-responsive element |  | |
| TCA-element | 1 | 1 | - | *cis-acting* element involved in salicylic acid responsiveness |  | |
| DRE core | 1 | - | 1 | Drought responsive element | stress response | |
| MBS | 6 | 6 | 4 | MYB binding site involved in drought-inducibility |  | |
| LTR | - | - | 3 | *cis-acting* element involved in low-temperature responsiveness |  | |
| STRE | 4 | 4 | 11 | stress response elements |  | |
| WRE3 | - | - | 5 | stress response elements |  | |
| WUN-motif | - | - | 1 | *cis-acting* element involved in wound-responsiveness |  | |
| CAT-box | - | - | 4 | *cis-acting* regulatory element related to meristem expression | plant growth and development | |
| GCN4_motif | - | - | 1 | *cis-regulatory* element involved in endosperm expression |  |  |
| O2-site | 1 | 1 | 1 | *cis-acting* regulatory element involved in zein metabolism regulation |  |  |
